# Source-Resolved Wastewater Metagenomics Reveals Distinct Resistome and Virulome Landscapes Across an Urban Wastewater Continuum

**DOI:** 10.3390/antibiotics15090817

**Published:** 2026-08-23

**Authors:** Douha Shouqair, Subham Verma, Rashed Alghafri, Rania Nassar, Lobna Mohamed, Fatima Al Dhaheri, Dean Everett, Ahmed A. Shibl, Jorge Rodríguez, Danesh Moradigaravand, Mushtaq Khan, Richard Goering, Abiola Senok

**Affiliations:** 1 College of Medicine, Mohammed Bin Rashid University of Medicine and Health Sciences, Dubai P.O. Box 505055, United Arab Emirates; douha.shouqair@students.mbru.ac.ae (D.S.); subham.verma@dubaihealth.ae (S.V.); rania.nassar@dubaihealth.ae (R.N.); lobna.mohamed@dubaihealth.ae (L.M.); 2 Center for Microbial Sciences, Mohammed Bin Rashid University of Medicine and Health Sciences, Building 14, Dubai Healthcare City, Dubai P.O. Box 505055, United Arab Emirates; 3 General Department of Forensic Science and Criminology, Dubai Police, Dubai P.O. Box 1493, United Arab Emirates; r.alghafri@dubaipolice.gov.ae; 4 Department of Pediatrics, College of Medicine and Health Sciences, United Arab Emirates University, Al-Ain P.O. Box 15551, United Arab Emirates; fatimaald@uaeu.ac.ae; 5 Department of Public Health and Epidemiology, College of Medicine and Health Sciences, Khalifa University, Abu Dhabi P.O. Box 127788, United Arab Emirates; dean.everett@ku.ac.ae; 6 Biotechnology Center, Khalifa University, Abu Dhabi P.O. Box 127788, United Arab Emirates; 7 Infection Research Unit, Khalifa University, Abu Dhabi P.O. Box 127788, United Arab Emirates; 8 Public Health Research Center, New York University Abu Dhabi, Abu Dhabi P.O. Box 129188, United Arab Emirates; ahmed.shibl@nyu.edu; 9 Department of Chemical and P. Engineering, Khalifa University, Abu Dhabi P.O. Box 127788, United Arab Emirates; jorge.rodriguez@ku.ac.ae; 10 Laboratory of Infectious Disease Epidemiology, Biomedical Sciences (BioMed) Division, King Abdullah University of Science and Technology (KAUST), Thuwal 23955-6900, Saudi Arabia; danesh.moradigaravand@kaust.edu.sa; 11 Department of Medical Microbiology and Immunology, College of Medicine and Health Sciences, United Arab Emirates University, Al-Ain P.O. Box 15551, United Arab Emirates; mushtaq.khan@uaeu.ac.ae; 12 Zayed Bin Sultan Center for Health Sciences, United Arab Emirates University, Al-Ain P.O. Box 15551, United Arab Emirates; 13 Department of Medical Microbiology and Immunology, Creighton University School of Medicine, Omaha, NE 68178, USA; richardgoering@creighton.edu; 14 School of Dentistry, Cardiff University, Cardiff CF14 4XY, UK

**Keywords:** antimicrobial resistance, wastewater, shotgun metagenomics, One Health, WWTP, resistome, virulome

## Abstract

Background/Objectives: Wastewater-based antimicrobial resistance (AMR) surveillance typically relies on treatment plant influent as a single integrated matrix, obscuring source-specific signals. In arid settings where treated effluent is reused, understanding how resistomes and virulomes are structured across wastewater compartments is essential for One Health monitoring. Methods: Shotgun metagenomic sequencing was applied to 57 wastewater samples collected in Dubai, United Arab Emirates, between October 2024 and January 2025. Samples represented nine community sewer nodes, two tertiary-care hospital outflows, and influent and effluent from two wastewater treatment plants (WWTP). Datasets were used for taxonomic, resistome, and virulome profiling. Alpha diversity was compared using Wilcoxon rank-sum tests, beta-diversity differences were assessed using permutational multivariate analysis of variance, and source-associated AMR genes were identified using linear discriminant analysis effect size analysis. Results: A total of 1470 bacterial species, 822 antimicrobial resistance genes (ARGs), and 1554 virulence factor genes were identified. Bacterial diversity was significantly lower in WWTP effluent than in other compartments. Hospital wastewater was enriched for class D β-lactamases, including multiple *bla*_OXA_ variants, whereas community wastewater and WWTP influent shared dominant macrolide and aminoglycoside resistance genes including *msr*(*E*), *mph*(*E*), *strB* and *aadA1*. Despite marked reductions in bacterial diversity after treatment, no significant difference in ARG diversity was observed between WWTP influent and WWTP effluent (*p* = 0.558), with resistance genes such as *bla*_VEB_, *msr*(*E*), *mph*(*E*) detected in the latter. Virulome profiles shifted from fimbrial gene dominance in untreated sources toward biofilm- and persistence-associated genes in WWTP effluent. ARG alpha diversity varied over time, whereas taxonomic and virulome diversity remained stable. Conclusions: Community and influent wastewater capture population-level AMR carriage, hospital outflows concentrate clinically relevant resistance determinants, and WWTP effluent retains resistance markers despite microbial biomass reduction. Compartment-resolved metagenomic surveillance provides a practical One Health framework for identifying high-value monitoring points.

## 1. Introduction

Antimicrobial resistance (AMR) is one of the defining health threats of the twenty-first century, with an estimated 1.27 million deaths attributed to bacterial AMR globally in 2019 [1]. However, in most settings, surveillance remains largely organized around clinical detection, even though AMR is sustained by connected human and environmental systems [2]. Across these interconnected niches, resistant microorganisms, antimicrobial resistance genes (ARGs), virulence factor genes (VFGs) and mobile genetic elements persist and spread [3,4]. In this context, wastewater-based surveillance has become an important approach for tracking population-level dynamics of infectious diseases [5]. Urban wastewater pools microbial and genetic material from households, healthcare facilities and the wider community into a single readout, while functioning as a One Health interface for environmental AMR surveillance [6].

Within wastewater systems, ARGs are not only detected as isolated markers but form part of a broader resistome that may be maintained and disseminated through the wastewater mobilome [7,8]. Mobile genetic elements including plasmids, integrons, transposons, insertion sequences and phage-associated elements, can facilitate horizontal gene transfer between bacterial hosts [9,10]. The high microbial density and diversity of wastewater, together with residual antimicrobials, disinfectants, metals and other selective pressures, may therefore support the persistence and movement of clinically relevant resistance determinants. This makes wastewater an important environmental reservoir and potential transmission hub for AMR, in addition to its role as a surveillance matrix [7,8].

However, urban wastewater is not a homogeneous surveillance matrix. It includes community wastewater, which reflects population-level microbial carriage and antimicrobial exposure, and hospital wastewater, which concentrates clinically relevant organisms and resistance genes under sustained antimicrobial selection [11]. At wastewater treatment plants (WWTPs), these streams converge with broader city-scale inputs, diluting source-specific signals while generating a biologically complex influent. Treatment at WWTPs reduces microbial biomass and restructures community composition but can retain, enrich or selectively filter ARGs and VFGs in treated effluent [12,13]. This is consequential in arid and rapidly urbanizing regions, where WWTP effluent is increasingly reused for irrigation, landscaping and other non-potable applications [14]. In these settings, WWTP effluent is not only the endpoint of treatment; it may represent a route through which residual microbial communities, resistance determinants, and virulence-associated genes are reintroduced into the environment [15]. Despite this relevance, there remains a paucity of data on wastewater-based AMR surveillance in the Arabian Gulf region where rapid urbanization, high population mobility and expanding water reuse make it a distinctive setting for environmental AMR monitoring [16].

Analytical approaches for wastewater-based AMR surveillance include targeted molecular assays and untargeted sequencing approaches [17]. Targeted methods such as quantitative PCR are sensitive, relatively low-cost and effective for monitoring predefined organisms or resistance determinants, but they are constrained by prior target selection and provide limited insight into the broader microbial and functional context of complex wastewater communities [18]. However, shotgun metagenomics sequencing provides a hypothesis-independent, agnostic approach for gene-level profiling, enabling characterization of microbial community structure, ARGs, and VFGs in wastewater samples [19]. This broader resolution is particularly suited to compartment-resolved surveillance, enabling comparison of microbial communities, resistomes and virulomes across wastewater sources.

In this study, we applied shotgun metagenomic sequencing to wastewater sampled from community sewer nodes, tertiary-care hospital outflows, and WWTP influent and effluent in Dubai, United Arab Emirates (UAE), to characterize how microbial communities, resistomes, and virulomes are structured across distinct compartments of an arid urban system. By integrating taxonomic, ARG and VFG profiling, this approach supports a compartment-resolved interpretation of wastewater-based AMR surveillance.

## 2. Results

A total of 60 wastewater samples comprising 36 community wastewater samples from nine sewer nodes, eight hospital wastewater samples from two tertiary-care hospitals, eight WWTP influent samples and eight WWTP effluent samples were obtained. Three WWTP effluent samples were excluded due to insufficient DNA yield for reliable shotgun metagenomic sequencing, hence 57 metagenomes were included in the analysis. Median sequencing depth was 232.63 million paired-end reads (IQR: 174.62–306.15 million); ranging from: 11.11 to 560.55 million reads.

**Figure 1:**
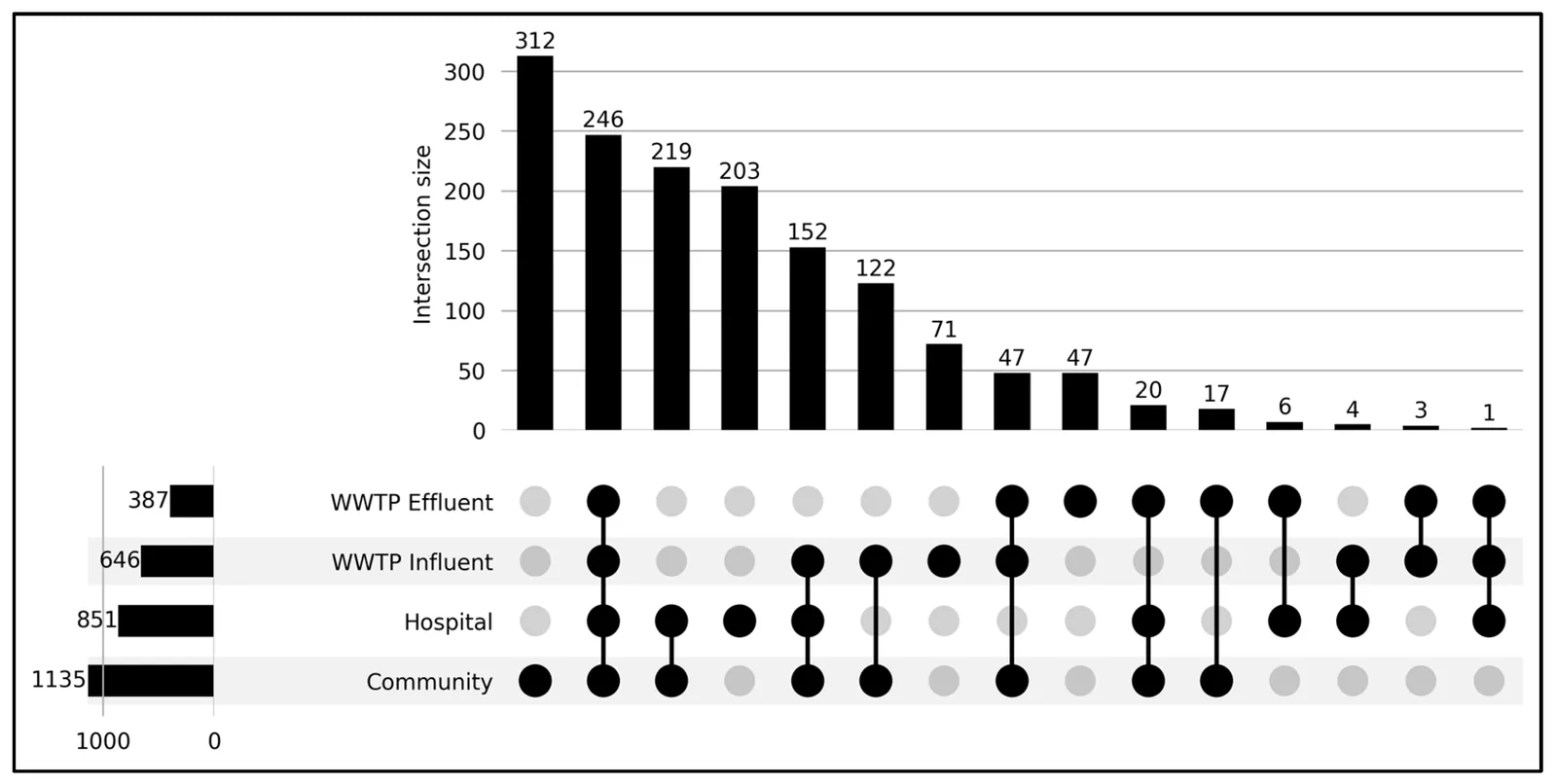
Distribution of bacterial species across wastewater sources. Black circles indicate sources included in each intersection, grey circles indicate sources not included, and connecting lines denote shared intersections.

Microbial community composition: Taxonomic profiling identified 1470 bacterial species across all wastewater samples. Cumulative species richness was highest in community wastewater (1135 species), followed by hospital (851), WWTP influent (646) and WWTP effluent (387). Median species richness per sample was 392.5 (interquartile range (IQR) 326.5–442) in hospital wastewater, 353 (286–397) in community wastewater, 258.5 (205–352.5) in WWTP influent and 142 (84.5–254.5) in WWTP effluent. Across all sources, 246 species were shared among the four groups (Figure 1).

**Figure 2:**
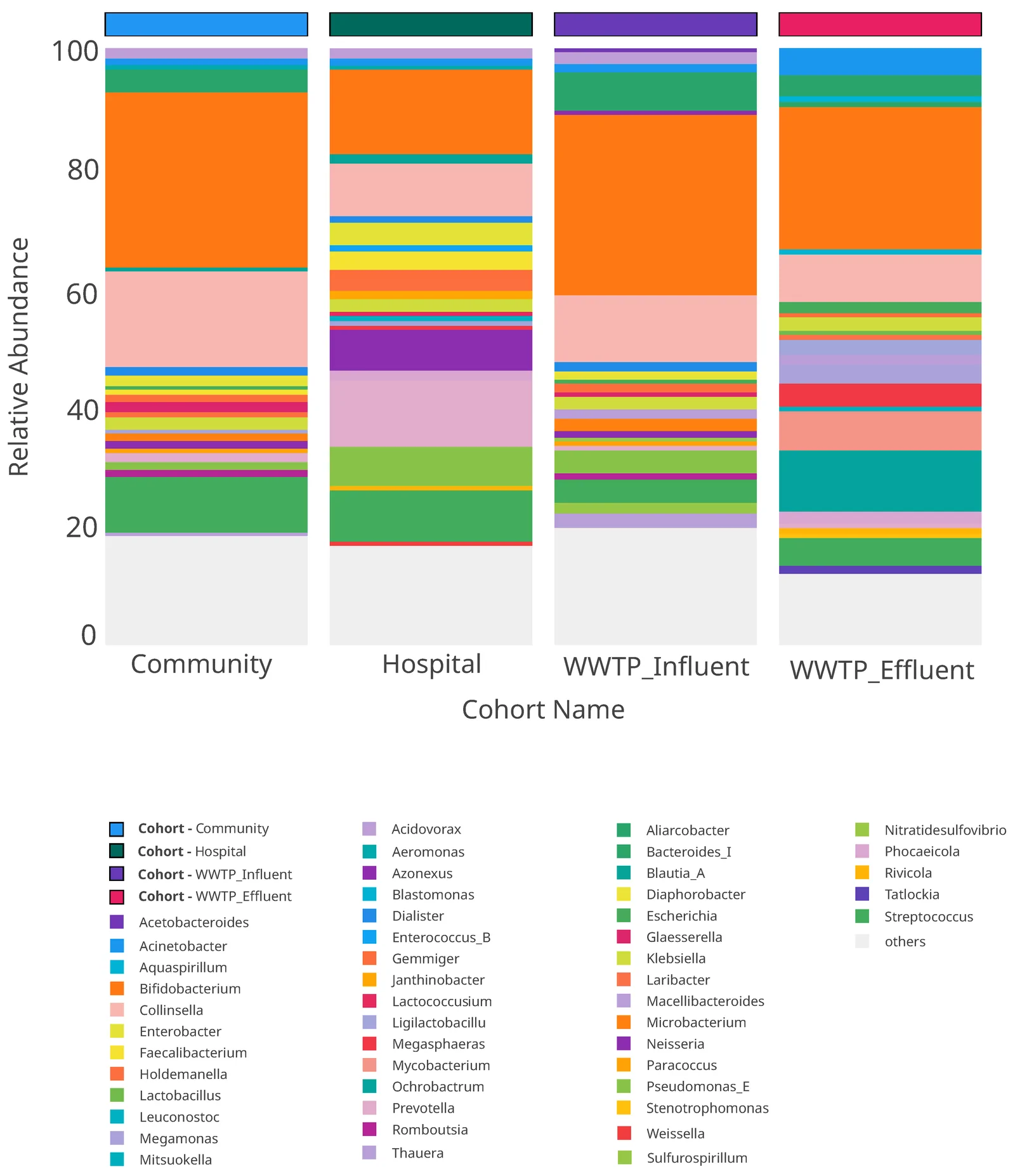
Genus-level taxonomic composition of the top 25 genera across wastewater sources.

Wastewater communities were dominated by Actinomycetota, Pseudomonadota, Bacillota, and Bacteroidota. In community wastewater, there was a predominance of *Bifidobacterium adolescentis* (11.92%) and *B. longum* (6.06%), with additional contributions from *Bifidobacterium* sp. 002742445 (3.53%) and *Aliarcobacter cryaerophilus_A* (2.38%). Hospital wastewater exhibited a distinct profile driven by *Neisseria suis* (6.84%), alongside *B. adolescentis* (5.26%) and *B. longum* (2.98%). WWTP influent closely resembled the community composition, with *B. adolescentis* (13.67%) and *B. longum* (5.64%) remaining dominant alongside *A. cryaerophilus_A* (3.71%). In contrast, WWTP effluent showed a shift in dominant taxa, characterized by *Ochrobactrum pseudintermedium* (9.48%), with contributions from *B. adolescentis* (8.23%) and *Mycobacterium fortuitum* (5.69%) (Figure 2). Several clinically relevant organisms were detected across wastewater sources and species-level relative abundances are provided in shown in Supplementary Figures S1 and S2. Differences in *Enterococcus faecium* and *Enterobacter* spp. were not statistically significant between compartments. In contrast, *Escherichia coli* was significantly enriched in WWTP effluent, whereas *Streptococcus* spp. showed higher relative abundances in community and hospital wastewater (Supplementary Figure S1).

**Figure 3:**
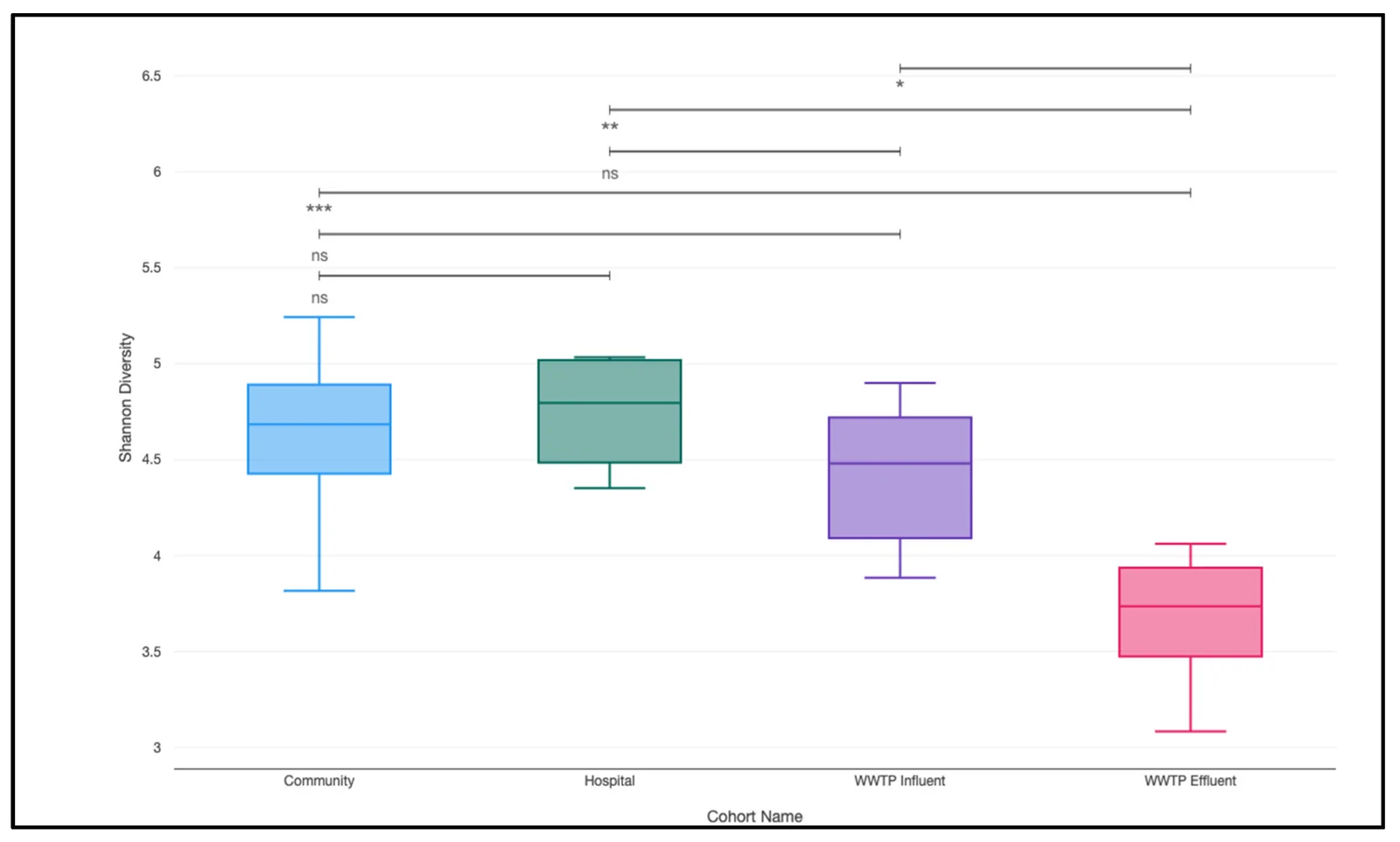
Alpha diversity of bacterial communities across cohorts measured by the Shannon index. Boxplots show the distribution of diversity in community, hospital, WWTP influent and WWTP effluent samples. Significance is indicated as * *p* < 0.05, ** *p* < 0.01, *** *p* < 0.001; ns indicates a non-significant difference.

Alpha diversity of bacterial communities varied across wastewater sources (Shannon index 3.74–4.80; Figure 3), with the highest values in hospital samples, followed by community and WWTP influent. Differences between these three sources were not statistically significant. WWTP effluent showed the lowest diversity, significantly lower than that of all other sources.

**Figure 4:**
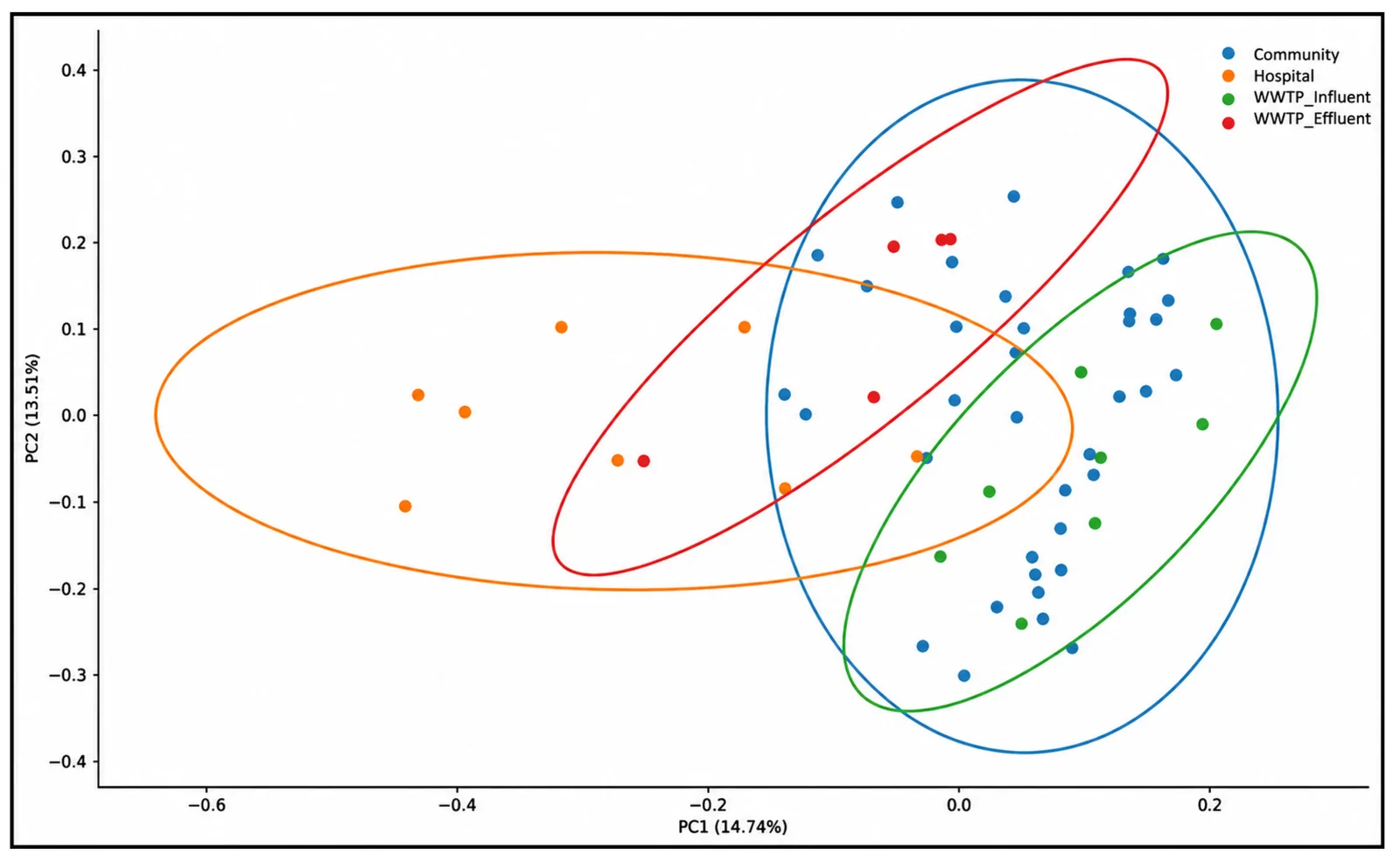
PCoA of Bray–Curtis dissimilarities of bacterial species based on relative abundance. Samples are coloured by cohort (community, hospital, WWTP influent and WWTP effluent). The first two principal coordinates explain 14.74% (PC1) and 13.51% (PC2) of the variance. Ellipses represent 95% confidence intervals around group centroids.

Principal coordinates analysis (PCoA) based on Bray–Curtis dissimilarities showed source-specific separation, with PC1 and PC2 explaining 14.74% and 13.51% of the variance (Figure 4). Hospital samples clustered separately, whereas community and WWTP influent samples partially overlapped; wastewater effluent samples were more dispersed with limited overlap. Permutational multivariate analysis of variance (PERMANOVA) confirmed a significant cohort effect (*p* = 0.001).

**Figure 5:**
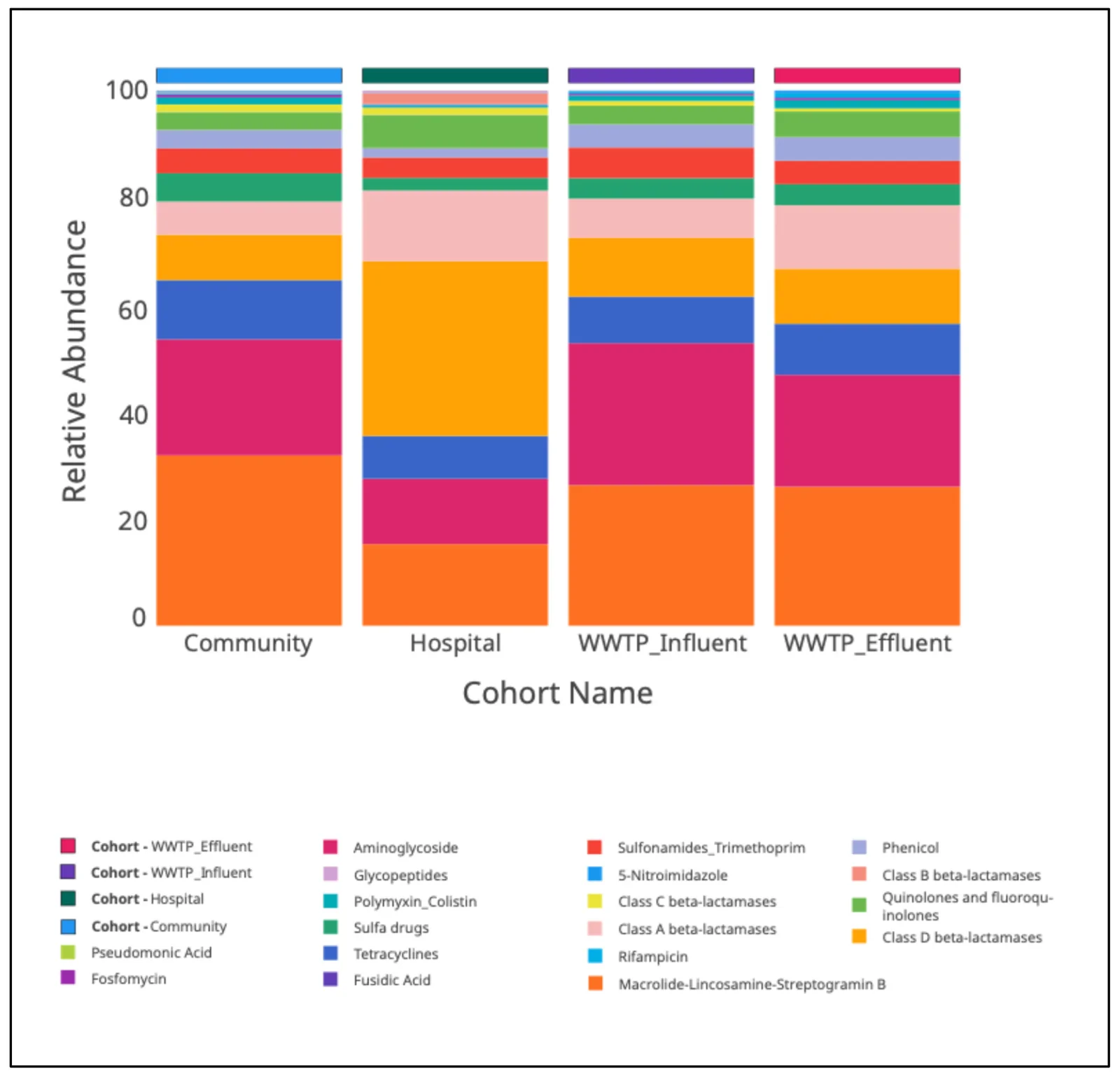
Relative abundance of ARGs and Antibiotic classes with ARGs across wastewater sources.

Antimicrobial resistance genes (ARGs): Resistome profiling across the metagenomes identified 822 ARGs, with the highest number detected in community wastewater (*n* = 716), followed by hospital wastewater (*n* = 436), WWTP influent (*n* = 404) and WWTP effluent (*n* = 326) (Figure 5). ARGs spanning multiple antimicrobial classes were detected across all sources, including macrolide–lincosamide–streptogramin B (MLSB), aminoglycosides, β-lactams, tetracyclines and sulfonamides resistance genes. Figure 5 shows ARGs and antibiotic classes with ARGs across wastewater sources.

Community wastewater was dominated by macrolide and aminoglycoside resistance genes, with *msr*(*E*) and *mph*(*E*) as the most abundant markers, followed by *strB* and *aadA1* with median relative abundances (IQR) of 9.62 (7.46–11.60), 8.80 (6.66–11.04), 3.04 (2.67–3.98) and 2.22 (1.79–2.54), respectively. A similar profile was observed in WWTP influent, where *msr*(*E*) and *mph*(*E*) remained dominant, with median relative abundances (IQR) of 8.97 (6.19–9.72) and 8.56 (5.64–9.10), respectively, alongside increased contributions from *strB* [3.75 (3.12–6.15)] and *aadA1* [2.93 (2.59–3.45)], and persistent detection of *bla*_LCR_ [2.02 (1.45–2.22)]. In contrast, hospital wastewater exhibited a distinct resistome characterized by significant enrichment of class D β-lactamases, which reached 32.63% relative abundance and were significantly higher than in community wastewater, WWTP influent, and WWTP effluent (*p* < 0.001, *p* < 0.01, and *p* < 0.01, respectively). Detected variants included *bla*_OXA-233_, *bla*_OXA-16_, *bla*_OXA-454_, *bla*_OXA-368_, and *bla*_OXA-251_.

Hierarchical clustering of ARG relative abundances showed no clear separation between WWTP influent and effluent samples (Supplementary Figure S3), although influent samples displayed slightly higher overall relative abundances across multiple ARGs. Key ARGs shared between influent and effluent included the *msr*(*E*), *mph*(*E*), *strB* and *aadA*. WWTP influent samples showed enrichment of *sul1*, whereas WWTP effluent samples featured β-lactamase genes such as *bla*_VEB-3_ and *bla*_VEB-6_. Median relative abundances were 0.09 (0.05–3.95), 2.28 (1.76–4.83) and 3.95 (2.25–5.66), respectively. ARG diversity, as measured by the Shannon index, did not differ significantly between WWTP influent and effluent samples (*p* = 0.558).

**Figure 6:**
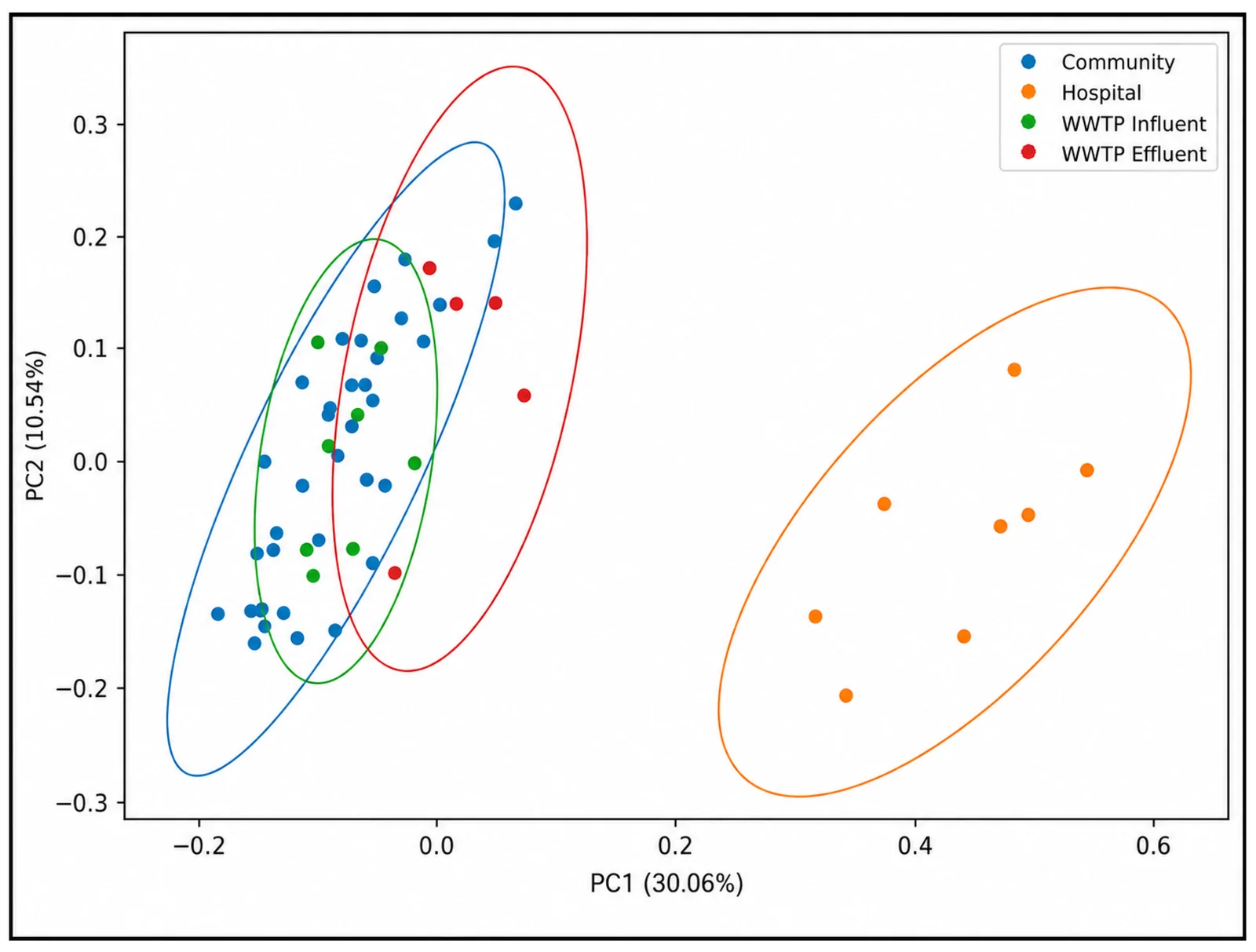
Bray–Curtis PCoA of ARG profiles across wastewater sources. Points represent individual samples coloured by source, with ellipses indicating 95% confidence intervals.

Bray–Curtis PCoA showed significant source-associated differences in resistome composition (*p* = 0.001; Figure 6). Hospital samples formed a distinct cluster, whereas community and WWTP influent samples overlapped substantially. The WWTP effluent samples were more dispersed and partially overlapped with non-hospital groups.

Linear discriminant analysis effect size (LefSe) analysis further resolved the ARGs driving source-associated resistome differences (LDA > 3.75; Supplementary Figure S4). Hospital wastewater was distinguished by class D β-lactamases, including *bla*_OXA_ variants, together with *qnrD1*, whereas community wastewater and WWTP influent were characterized by macrolide and aminoglycoside resistance determinants. Effluent-associated resistance determinants included persistent macrolide and aminoglycoside genes, alongside the chloramphenicol resistance gene *cmlA1*.

**Figure 7:**
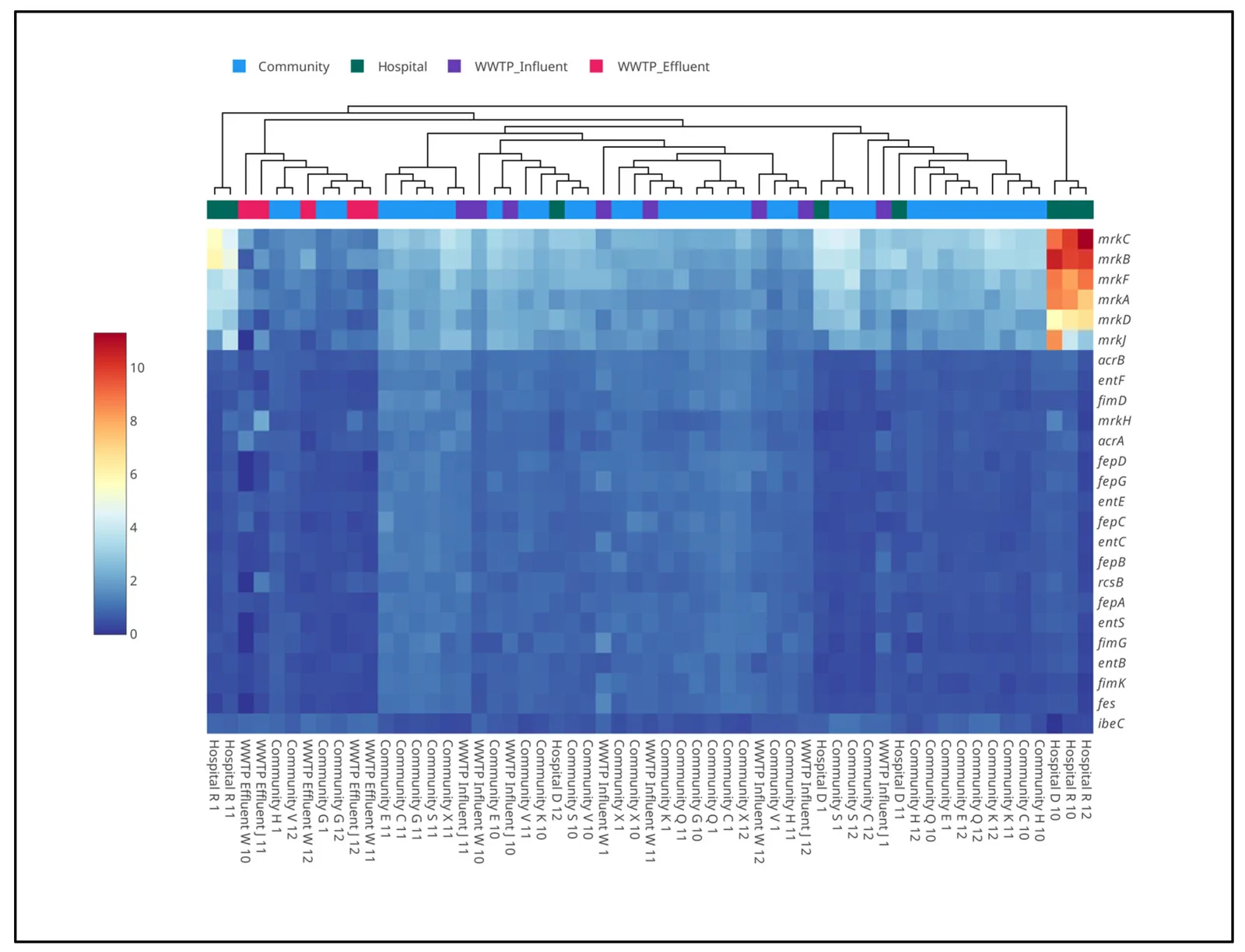
Relative abundances of VFGs across wastewater samples, with hierarchical clustering of samples.

Virulence factor genes (VFGs): VFGs profiling identified 1554 genes and revealed source-specific virulome patterns. Community, hospital and WWTP influent samples were dominated by the fimbrial operon genes *mrkC*, *mrkB*, *mrkF*, *mrkA*, *mrkD* and *mrkJ*. These had comparable abundance in community wastewater and WWTP influent, but were significantly enriched in hospital wastewater (*p* < 0.05). Additional virulence genes, including *fimD* and the iron acquisition genes *entF* and *fepD*, were also detected in these samples, but at lower abundance. In contrast, WWTP effluent showed reduced overall VFGs abundance and a distinct profile with enrichment of the lipid A acyltransferase gene *acpXL* (median relative abundance [IQR]; 11.73 [0.08–23.37]). WWTP effluent samples also exhibited increased abundance of curli-associated genes (*csgE*, *csgB* and *csgD*), while *mrk* genes remained detectable (Figure 7).

**Figure 8:**
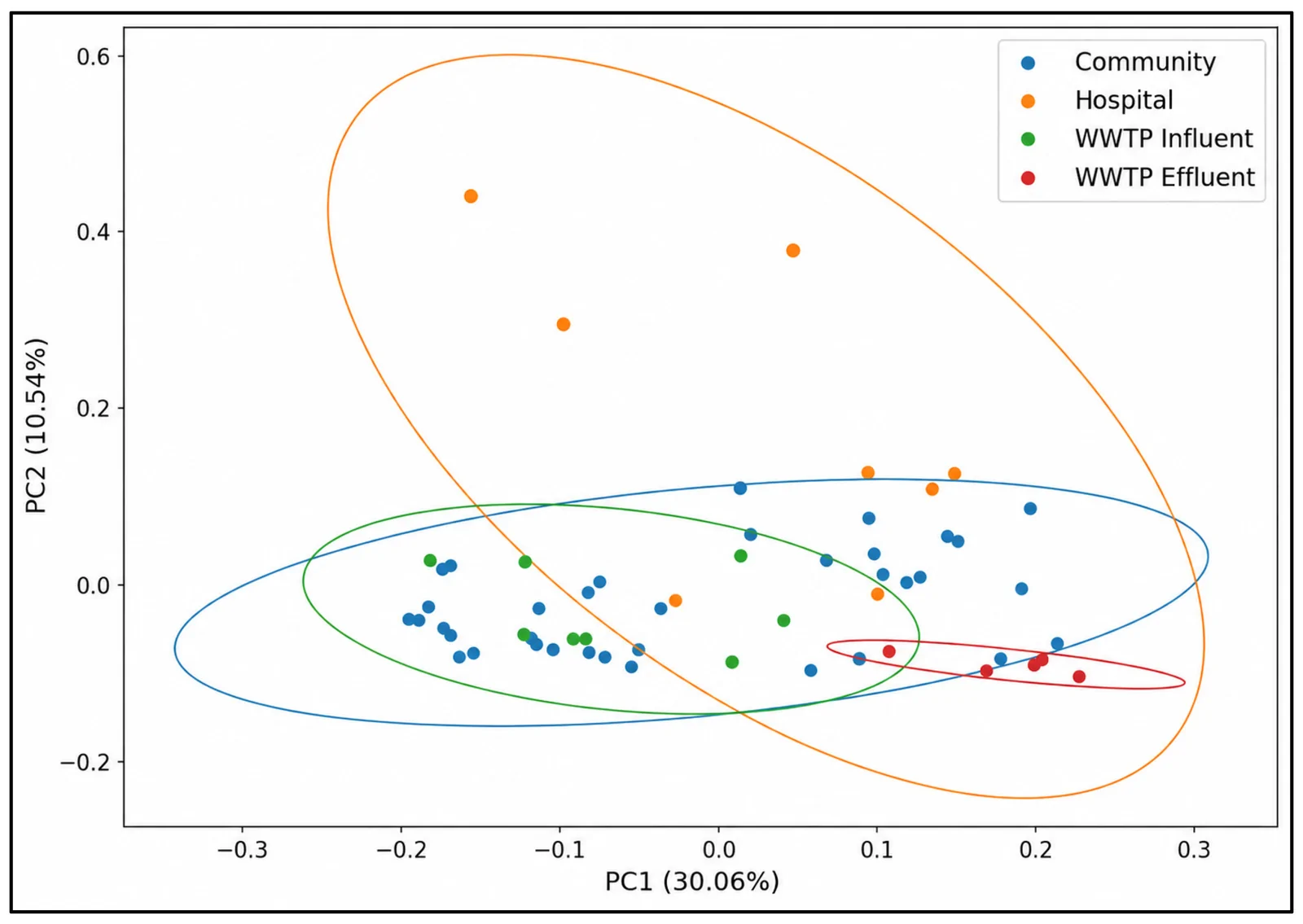
Bray–Curtis PCoA of VFG profiles across wastewater sources. Points represent the individual samples coloured by source, and ellipses indicate 95% confidence intervals.

PCoA showed significant source-associated differences in virulome composition (PERMANOVA, *p* = 0.001; Figure 8). Hospital wastewater formed a distinct and more dispersed cluster, whereas community wastewater and WWTP influent overlapped substantially and did not differ significantly (*p* = 0.201). WWTP effluent samples clustered more tightly, with limited overlap with the other sources (Figure 8).

Across all 57 samples, ARGs and VFGs Bray–Curtis dissimilarities matrices were significantly positively correlated by Mantel tests using both Pearson and Spearman coefficients (Pearson *r* = 0.58, *p* = 0.001; Spearman ρ = 0.59, *p* = 0.001).

Alpha diversity (Shannon index) was assessed across taxonomic, resistome, and virulome profiles over the sampling periods. Taxonomic and VFG diversity remained stable with no significant temporal variation observed. In contrast, ARG alpha diversity exhibited temporal variation with Wilcoxon Rank Sum pairwise comparisons revealing significant differences across most sampling months (*p* < 0.05; Supplementary Figure S5).

## 3. Discussion

The present study demonstrates that the urban wastewater continuum comprises distinct microbial, resistome, and virulome niches, each with its own ecological signature [20]. Community wastewater and WWTP influent reflected population-level carriage, hospital wastewater concentrated healthcare-associated resistance determinants, and WWTP effluent represented the residual biological footprint released after treatment [19,20]. Sampling these compartments separately revealed heterogeneity that is obscured when surveillance relies on WWTP influent samples alone [20]. This resolution is particularly relevant in settings with extensive treated wastewater reuse, where engineered water systems intersect with environmental and human health pathways, underscoring the importance of compartment-specific surveillance within a One Health framework [6,19].

Across the 57 metagenomes analyzed, bacterial richness was high throughout the wastewater network but partitioned distinctly by compartment. Community wastewater showed the highest cumulative richness, consistent with the breadth and heterogeneity of the residential catchments sampled, whereas hospital wastewater showed the highest median richness per sample, reflecting concentrated within-site complexity [21]. WWTP effluent had the lowest richness and Shannon diversity, indicating marked treatment-associated restructuring [19]. These patterns indicate that wastewater compartments are not interchangeable matrices, as each captures a biologically distinct surveillance signal. Community wastewater and WWTP influent were dominated by gut-associated taxa, notably *B. adolescentis* and *B. longum*, with an overlapping community structure [22]. Whilst this finding supports the use of WWTP influent as a broad urban composite, it underscores the added value of upstream community-node sampling for finer source resolution. In contrast, WWTP effluent composition was consistent with a selected residual community shaped by post-treatment biomass reduction [19].

Clinically relevant taxa, including ESKAPE-associated organisms, were detected across the wastewater continuum, demonstrating the capacity of metagenomic surveillance to identify clinically important microorganisms within complex mixed communities [19,23]. However, many of these taxa occurred at low relative abundance and showed limited compartment-specific enrichment. Their patterns were not uniformly source-specific, suggesting contributions from both community and healthcare inputs, as well as treatment-related shifts. This supports compartment-resolved surveillance, as reliance on a single wastewater matrix may obscure organism-specific enrichment and persistence across the urban wastewater system.

The resistome analysis provided the most direct AMR surveillance signal. Community wastewater and WWTP influent shared dominant macrolide and aminoglycoside resistance genes, including *msr*(*E*), *mph*(*E*), *strB*, and *aadA1*, indicating continuity between upstream catchment community inputs and WWTP influent profiles [24]. Hospital wastewater yielded a clinically enriched resistome signal, including significant enrichment of class D β-lactamase genes, and detection of *qnrD1*. As shotgun metagenomic sequencing detects genetic material rather than viable organisms or active gene expression, this finding is indicative of enrichment of clinically relevant resistance determinants rather than direct evidence of viable resistant pathogens or transmission risk [25]. This supports hospital wastewater as a high-yield sentinel matrix for clinically relevant resistance determinants before downstream dilution [26]. Importantly, this enrichment does not imply that hospitals are the sole source of AMR within urban wastewater systems. Rather, it indicates that hospital outflows act as concentrated reservoirs of healthcare-associated resistomes, whereas community wastewater and WWTP influent remain critical for capturing broader population-level signals. Although WWTP effluent had the lowest bacterial richness and diversity, ARG diversity did not differ significantly between influent and effluent samples. This suggests that microbial reduction did not translate into proportional reductions in the resistome profiles [27]. Residual ARGs may persist through surviving taxa, particle or biofilm-associated DNA, or compositional shifts caused by treatment or selective pressure [15,28]. The retention of selected macrolide and aminoglycoside resistance determinants and β-lactamase signals, including *bla*_VEB_ variants, identifies treated effluent as a critical endpoint for AMR monitoring. These findings do not imply inadequate treatment, as conventional WWTPs are not designed to eliminate ARGs or VFGs, rather, they define the residual AMR signal at the point where treated wastewater may reconnect with environmental pathways through discharge or reuse [29].

Virulome profiling provided important functional context to the resistome. Untreated wastewater compartments were dominated by fimbrial and adhesion-associated genes, particularly members of the *mrk* operon, with the strongest signals observed in hospital wastewater [30]. In contrast, WWTP effluent was enriched for *acpXL* and curli-associated genes, suggesting that the residual post-treatment community is shaped less by host-associated virulence traits and more by ecological characteristics that favour membrane adaptation, surface attachment, biofilm formation, and persistence in engineered and environmental habitats [28]. Although the detection of virulence factor genes does not imply gene expression, pathogenicity, or disease risk [31], these profiles provide insight into the functional attributes that may support microbial persistence and host–environment interactions within wastewater ecosystems. Temporal analysis added a dynamic monitoring layer. ARG alpha diversity varied significantly across the four-month sampling period, whereas taxonomic and virulome diversity remained comparatively stable. This suggests that resistome profiles may respond more rapidly than broad microbial community structure to short-term changes in antimicrobial use, healthcare activity, infection pressure, population mobility, or catchment inputs [32]. Although significant month-to-month differences in ARG diversity were observed, the four-month sampling window limits conclusions regarding broader temporal trends or seasonality. Therefore, these findings should be interpreted as short-term temporal variation within the study period, and longer longitudinal surveillance will be required to determine whether such patterns persist over time [24,33,34].

Collectively, these findings demonstrate the value of a layered metagenomic approach for AMR surveillance across the urban wastewater continuum. Taxonomic profiling resolved compartment-specific microbial communities, resistome analysis identified distinct resistance gene distributions, and virulome profiling provided complementary functional context. Temporal sampling further showed that ARG composition varied more across sampling periods than either taxonomic or virulome profiles. Together, these data reveal compartment-specific AMR signatures that would be obscured by WWTP influent-only surveillance.

Although shotgun metagenomics is a costly and technically demanding approach, it provides hypothesis-independent resolution that is particularly valuable during the discovery phase of wastewater-based AMR surveillance and in regions where baseline data are limited. Targeted approaches, such as high-throughput qPCR, can then be used as alternate options for routine or higher-frequency surveillance once key markers have been defined [25].

These findings should be interpreted within the limitations of the study. Community and hospital wastewater were collected as grab samples, whereas WWTP influent and effluent were collected as 24 h composites; this difference in sampling methodology may partly account for observed inter-compartment variation and should be considered alongside biological interpretations. To reduce the influence of short-term temporal variability, samples were collected repeatedly over a four-month study period rather than relying on a single sampling event.

More broadly, shotgun metagenomics detects genetic material rather than viability, gene expression, or transmission potential [35,36]. Consequently, the persistence of ARGs and virulence-associated genes in treated effluent reflects residual genetic signals at the point of environmental discharge rather than demonstrated exposure risk. Future studies integrating absolute quantification, viability-informed approaches, antibiotic residue measurements, mobile genetic element analysis, and linked clinical and treatment-process metadata would strengthen source attribution and risk assessment.

By resolving microbial, resistome, and virulome signatures across community wastewater, hospital wastewater, WWTP influent, and WWTP effluent, this study demonstrates the value of compartment-specific surveillance for understanding AMR dynamics across the wastewater continuum. Such an approach is particularly relevant in water-scarce settings where treated wastewater reuse creates close links between engineered water systems, environmental reservoirs, and human populations, reinforcing the role of wastewater surveillance within a One Health AMR monitoring framework [37].

## 4. Materials and Methods

Sampling framework: Wastewater was sampled monthly between October 2024 and January 2025 at 13 sites in Dubai, UAE, including nine community pumping stations, two tertiary-care hospital outflows, and two wastewater treatment plants (WWTPs) [25]. To reduce temporal variability, all sites were sampled within the same predefined monthly window, with a maximum interval of 48 h between collections. Community and hospital wastewater samples were obtained as grab samples, whereas WWTP influent and effluent were collected as 24 h flow-composite samples using refrigerated autosamplers (Hach, Loveland, CO, USA). For each sampling event, 1 L of wastewater was collected in sterile wide-mouth bottles (Azlon, Stone, Staffordshire, UK) and transported to the laboratory at 4 °C for downstream processing.

DNA extraction: Total community DNA was extracted using the DNeasy PowerSoil Pro Kit (QIAGEN, Hilden, Germany) according to the manufacturer’s instructions [38,39,40]. Purified DNA was washed, eluted, and quantified using a Qubit Flex fluorometer and the Qubit dsDNA High Sensitivity Assay Kit (Thermo Fisher Scientific, Waltham, MA, USA) [39].

Shotgun metagenomic sequencing: DNA libraries were prepared using the Watchmaker DNA Library Prep Kit with Fragmentation (7K0019-1K; Watchmaker Genomics, Boulder, CO, USA) according to CosmosID validated workflow (CosmosID, Germantown, MD, USA) [38,39]. Briefly, genomic DNA (1 ng) underwent enzymatic fragmentation, end repair, and A-tailing using a master mix of Watchmaker Frag/AT Buffer and Frag/AT Enzyme Mix, with incubation at 37 °C for 5 min followed by 65 °C for 30 min. IDT xGen Stubby Adapters were ligated to the processed DNA using the kit-supplied ligation reagents at 20 °C for 15 min. Adapter-ligated libraries were purified using CleanNGS magnetic beads (CleanNA, Waddinxveen, The Netherlands) and eluted in 10 mM Tris-HCl (pH 8.0). The IDT xGen UDI Primers and the kit-supplied amplification mix were then added, and the libraries were amplified for 16 PCR cycles. The amplified libraries were purified using CleanNGS magnetic beads and eluted in nuclease-free water. Final library concentrations were quantified using the Qubit fluorometer dsDNA High Sensitivity Assay (Thermo Fisher Scientific, Waltham, MA, USA). Prior to sequencing, libraries were converted for platform compatibility using the Adept workflow (Element Biosciences, San Diego, CA, USA) [38,39]. Paired-end sequencing (2 × 150 bp) was performed on an Element AVITI platform using Cloudbreak chemistry (Element Biosciences, San Diego, CA, USA) [38,39,40,41].

Bioinformatic analysis: Shotgun metagenomic data were analyzed using the CosmosID bioinformatics platform (v2.0; CosmosID, Germantown, MD, USA) for taxonomic, resistome, and virulome profiling. Taxonomic classification was performed using the Taxa-Kepler Domain workflow (v1.1.0; database v3.0.0), while resistome and virulome profiling were performed using the AMR and Virulence Marker workflow (v1.1.0; database v1.1.0). After quality filtering, reads were matched against curated reference databases using a k-mer-based algorithm for microbial classification and functional gene detection. Taxa were assigned to the species level when supported by sequence data and reference coverage, with strain-level resolution reported where applicable. Relative abundance values were calculated as reads per million and normalized to total prokaryotic reads. ARGs and VFGs were identified using the ResFinder and VFDB databases through the CosmosID Kepler-AMR/VF workflow.

Alpha diversity was assessed using the Shannon index and compared across cohorts using the Wilcoxon rank-sum test. Beta diversity was assessed using Bray–Curtis dissimilarity and visualized by principal coordinate analysis (PCoA), with statistical significance determined by PERMANOVA. To assess whether ARGs and VFGs profiles co-varied across the wastewater continuum, Mantel tests were performed between the Bray–Curtis dissimilarity matrices of ARGs and VFGs relative abundance profiles across all 57 samples. Both Pearson and Spearman correlation coefficients were calculated with 999 permutations to assess significance using Python SciPy v1.17.1. Linear discriminant analysis effect size (LEfSe) was performed on ARG profiles to assess differential abundance across wastewater sources. Statistical significance was set at *p* < 0.05, and an LDA score threshold of 3.75 was applied.

## 5. Conclusions

This study demonstrates that compartment-resolved wastewater metagenomics can reveal distinct microbial, resistome, and virulome signatures across the wastewater continuum. Community wastewater and WWTP influent captured broad population-level AMR signals, hospital wastewater highlighted clinically enriched resistance determinants, and treated effluent retained residual resistance and virulence-associated markers despite reduced bacterial diversity. These findings show that wastewater compartments are not interchangeable surveillance matrices and that influent-only monitoring may obscure source-specific AMR patterns.

## Data Availability

Raw metagenomic sequencing reads have been deposited in the NCBI Sequence Read Archive (SRA) under BioProject accession PRJNA1483809.

## Supplementary Materials

The following supporting information can be downloaded at: https://www.mdpi.com/article/10.3390/antibiotics15090817/s1.

## Abbreviations

The following abbreviations are used in this manuscript:

AMR	Antimicrobial resistance
ARG	Antimicrobial resistance gene
bp	Base pair
CRI	Collaborative Research Initiative
DNA	Deoxyribonucleic acid
ESKAPE	*Enterococcus faecium*, *Staphylococcus aureus*, *Klebsiella pneumoniae*, *Acinetobacter baumannii*, *Pseudomonas aeruginosa*, and *Enterobacter* spp.
IDT	Integrated DNA Technologies
IQR	Interquartile range
LDA	Linear discriminant analysis
LEfSe	Linear discriminant analysis effect size
MGE	Mobile genetic element
MLSB	Macrolide–lincosamide–streptogramin B
NCBI	National Center for Biotechnology Information
NIH	National Institutes of Health
PCoA	Principal coordinates analysis
PCR	Polymerase chain reaction
PERMANOVA	Permutational multivariate analysis of variance
qPCR	Quantitative polymerase chain reaction
RNA	Ribonucleic acid
SRA	Sequence Read Archive
UAE	United Arab Emirates
UDI	Unique dual index
VFDB	Virulence Factor Database
VFG	Virulence factor gene
WWTP	Wastewater treatment plant

## References

[1] Murray C.J.L., Ikuta K.S., Sharara F., Swetschinski L., Aguilar G.R., Gray A., Han C., Bisignano C., Rao P., Wool E. (2022). Global burden of bacterial antimicrobial resistance in 2019: A systematic analysis. Lancet.

[2] World Health Organization (2022). Global Antimicrobial Resistance and Use Surveillance System (GLASS) Report 2022.

[3] Feng Y., Lu X., Zhao J., Li H., Xu J., Li Z., Wang M., Peng Y., Tian T., Yuan G. (2025). Regional antimicrobial resistance gene flow among the One Health sectors in China. Microbiome.

[4] Berglund F., Ebmeyer S., Kristiansson E., Larsson D.G.J. (2023). Evidence for wastewaters as environments where mobile antibiotic resistance genes emerge. Commun. Biol..

[5] Kilaru P., Hill D., Anderson K., Collins M.B., Green H., Kmush B.L., Larsen D.A. (2023). Wastewater surveillance for infectious disease: A systematic review. Am. J. Epidemiol..

[6] Punch R., Azani R., Ellison C., Majury A., Hynds P.D., Payne S.J., Brown R.S. (2025). The surveillance of antimicrobial resistance in wastewater from a One Health perspective: A global scoping and temporal review (2014–2024). One Health.

[7] Olsen N.S., Riber L. (2025). Metagenomics as a Transformative Tool for Antibiotic Resistance Surveillance: Highlighting the Impact of Mobile Genetic Elements with a Focus on the Complex Role of Phages. Antibiotics.

[8] Uluseker C., Kaster K.M., Thorsen K., Basiry D., Shobana S., Jain M., Kumar G., Kommedal R., Pala-Ozkok I. (2021). A Review on Occurrence and Spread of Antibiotic Resistance in Wastewaters and in Wastewater Treatment Plants: Mechanisms and Perspectives. Front. Microbiol..

[9] Tokuda M., Shintani M. (2024). Microbial Evolution through Horizontal Gene Transfer by Mobile Genetic Elements. Microb. Biotechnol..

[10] Kumavath R., Gupta P., Tatta E.R., Mohan M.S., Salim S.A., Busi S. (2025). Unraveling the Role of Mobile Genetic Elements in Antibiotic Resistance Transmission and Defense Strategies in Bacteria. Front. Syst. Biol..

[11] Lan L., Wang Y., Chen Y., Wang T., Zhang J., Tan B. (2025). A review on the prevalence and treatment of antibiotic resistance genes in hospital wastewater. Toxics.

[12] Garner E., Maile-Moskowitz A., Angeles L.F., Flach C.-F., Aga D.S., Nambi I., Larsson D.G.J., Bürgmann H., Zhang T., Vikesland P.J. (2024). Metagenomic profiling of internationally sourced sewage influents and effluents yields insight into selecting targets for antibiotic resistance monitoring. Environ. Sci. Technol..

[13] Li Z., Guo X., Liu B., Huang T., Liu R., Liu X. (2024). Metagenome sequencing reveals shifts in phage-associated antibiotic resistance genes from influent to effluent in wastewater treatment plants. Water Res..

[14] Florides F., Giannakoudi M., Ioannou G., Lazaridou D., Lamprinidou E., Loukoutos N., Spyridou M., Tosounidis E., Xanthopoulou M., Katsoyiannis I.A. (2024). Water reuse: A comprehensive review. Environments.

[15] Drane K., Sheehan M., Whelan A., Ariel E., Kinobe R. (2024). The role of wastewater treatment plants in dissemination of antibiotic resistance: Source, measurement, removal and risk assessment. Antibiotics.

[16] Alghamdi B., Albedah N., Almalki T., Almudarra S., Penttinen P., Wang C., Hong P.-Y. (2026). Wastewater-based surveillance of microbial pathogens in GCC countries (2015–2025): A scoping review and questionnaire survey with stakeholders. Front. Public Health.

[17] Knight M.E., Webster G., Perry W.B., Baldwin A., Rushton L., Pass D.A., Cross G., Durance I., Muziasari W., Kille P. (2024). National-scale antimicrobial resistance surveillance in wastewater: A comparative analysis of HT qPCR and metagenomic approaches. Water Res..

[18] Taylor W., Bohm K., Dyet K., Weaver L., Pattis I. (2025). Comparative analysis of qPCR and metagenomics for detecting antimicrobial resistance in wastewater: A case study. BMC Res. Notes.

[19] Chen W.Y., Lee C.P., Pavlović J., Pangallo D., Wu J.H. (2024). Characterization of microbiome, resistome, mobilome, and virulome in anoxic and oxic wastewater treatment processes in Slovakia and Taiwan. Heliyon.

[20] Ramos B., Lourenço A.B., Monteiro S., Santos R., Cunha M.V. (2024). Metagenomic profiling of raw wastewater in Portugal highlights microbiota and resistome signatures of public health interest beyond the usual suspects. Sci. Total Environ..

[21] Silvester R., Perry W.B., Webster G., Rushton L., Baldwin A., Pass D.A., Healey N., Farkas K., Craine N., Cross G. (2025). Metagenomics unveils the role of hospitals and wastewater treatment plants on the environmental burden of antibiotic resistance genes and opportunistic pathogens. Sci. Total Environ..

[22] Resnick I.G., Levin M.A. (1981). Assessment of bifidobacteria as indicators of human fecal pollution. Appl. Environ. Microbiol..

[23] Marutescu L.G., Popa M., Gheorghe-Barbu I., Barbu I.C., Rodríguez-Molina D., Berglund F., Blaak H., Flach C.-F., Kemper M.A., Spießberger B. (2023). Wastewater treatment plants, an “escape gate” for ESCAPE pathogens. Front. Microbiol..

[24] Munk P., Brinch C., Møller F.D., Petersen T.N., Hendriksen R.S., Seyfarth A.M., Kjeldgaard J.S., Svendsen C.A., van Bunnik B., Berglund F. (2022). Genomic analysis of sewage from 101 countries reveals global landscape of antimicrobial resistance. Nat. Commun..

[25] Shouqair D., Alghafri R., Verma S., Naji M., Albastaki A., Al Dhaheri F., Hachim M.Y., Nassar R., Shibl A.A., Rodríguez J. (2026). Antimicrobial resistance across the urban wastewater continuum: A One Health assessment using high-throughput qPCR. Antibiotics.

[26] Lymperatou D., Konstantopoulou R., Mentsis M., Atzemoglou N., Diamanti C., Tzourtzos I., Naka K.K., Mitsis M., Konstantina G., Milionis H. (2025). Hospital wastewater surveillance and antimicrobial resistance: A narrative review. Microorganisms.

[27] Liu H., Li Z., Liu C., Qiang Z., Karanfil T., Yang M. (2022). Elimination and redistribution of intracellular and extracellular antibiotic resistance genes in water and wastewater disinfection processes: A review. ACS ES T Water.

[28] Haenelt S., Richnow H.-H., Müller J.A., Musat N. (2023). Antibiotic resistance indicator genes in biofilm and planktonic microbial communities after wastewater discharge. Front. Microbiol..

[29] Alam M.U., Ferdous S., Ercumen A., Lin A., Kamal A., Luies S.K., Sharior F., Khan R., Rahman Z., Parvez S.M. (2021). Effective treatment strategies for the removal of antibiotic-resistant bacteria, antibiotic-resistance genes, and antibiotic residues in the effluent from wastewater treatment plants receiving municipal, hospital, and domestic wastewater: Protocol for a systematic review. JMIR Res. Protoc..

[30] Mabeo O.R., van Niekerk B., Olanrewaju O.S., Bezuidenhout C.C., Molale-Tom L.G. (2025). Comprehensive genome analysis of MDR *Klebsiella pneumoniae* in influent and effluent of a selected wastewater treatment plant. Sci. Rep..

[31] Carneiro J., Pascoal F., Semedo M., Pratas D., Tomasino M.P., Rego A., Carvalho M.d.F., Mucha A.P., Magalhães C. (2023). Mapping human pathogens in wastewater using a metatranscriptomic approach. Environ. Res..

[32] Yin X., Yang Y., Deng Y., Huang Y., Li L., Chan L.Y., Zhang T. (2022). An assessment of resistome and mobilome in wastewater treatment plants through temporal and spatial metagenomic analysis. Water Res..

[33] Li W., Mao F., Ng C., Jong M.C., Goh S.G., Charles F.R., Ng O.T., Marimuthu K., He Y., Gin K.Y.-H. (2022). Population-based variations of a core resistome revealed by urban sewage metagenome surveillance. Environ. Int..

[34] Elder F.C.T., Proctor K., Barden R., Gaze W.H., Snape J., Feil E.J., Kasprzyk-Hordern B. (2021). Spatiotemporal profiling of antibiotics and resistance genes in a river catchment: Human population as the main driver of antibiotic and antibiotic resistance gene presence in the environment. Water Res..

[35] Dai D., Brown C., Bürgmann H., Larsson D.G.J., Nambi I., Zhang T., Flach C.-F., Pruden A., Vikesland P.J. (2022). Long-read metagenomic sequencing reveals shifts in associations of antibiotic resistance genes with mobile genetic elements from sewage to activated sludge. Microbiome.

[36] Shen J., McFarland A.G., Young V.B., Hayden M.K., Hartmann E.M. (2021). Toward accurate and robust environmental surveillance using metagenomics. Front. Genet..

[37] Gomes A., López-Cañizares J., Moreno-Candel M., Martinez-Alonso A., Allende A., Truchado P. (2026). Impact of treated wastewater reuse in agriculture on the transfer of antimicrobial-resistant bacteria and genes to edible crops: A One Health perspective. Front. Microbiol..

[38] Ayilaran E., McHugh O., Jung Y. (2025). Metagenomic sequencing dataset of microbial communities in onion and cabbage microgreens across substrates, *Salmonella* inoculation, and bacteriophage application. Data Brief.

[39] Frame L.A., Warren A., Al Qalam A., Corr P.G., Farah M., Karam M., Rangoussis K., Fahim Devin M., Celikkol Z., Gordon L. (2026). Brain health and the gut microbiome (bMicrobiome Study): A proof-of-concept, feasibility study integrating shotgun metagenomics, metrology, and multidimensional phenotyping across the cognitive aging spectrum. Gut Microbes Rep..

[40] Mahmud M.R., Uddin M.K., Kareljärvi P., Jalasvuori M., Peräkylä J., Eklund T., Biström M., Hasan S., Vatanen T., Kiljunen S. (2026). Impact of phage therapy in post-weaning piglets challenged with ETEC strain in a controlled minitrial. Porc. Health Manag..

[41] Nelon J.N., Eltaher S.S., Abdelhamid A.G. (2026). Shotgun metagenomic and phenotypic characterization of indigenous lactic acid bacteria from raw milk artisanal cheeses: Metagenomic functional insight and starter culture traits. Front. Microbiol..

