# Supplementary material for: Source-Resolved Wastewater Metagenomics Reveals Distinct Resistome and Virulome Landscapes Across an Urban Wastewater Continuum
Source: Antibiotics (Basel). 2026 Aug 23;15(9):817. doi: 10.3390/antibiotics15090817 (PMC13604000; doi:10.3390/antibiotics15090817)
Supplement: Supplementary file 1 [file antibiotics-15-00817-s001.zip › antibiotics-4438435-supplementary.pdf]

Supplementary Material

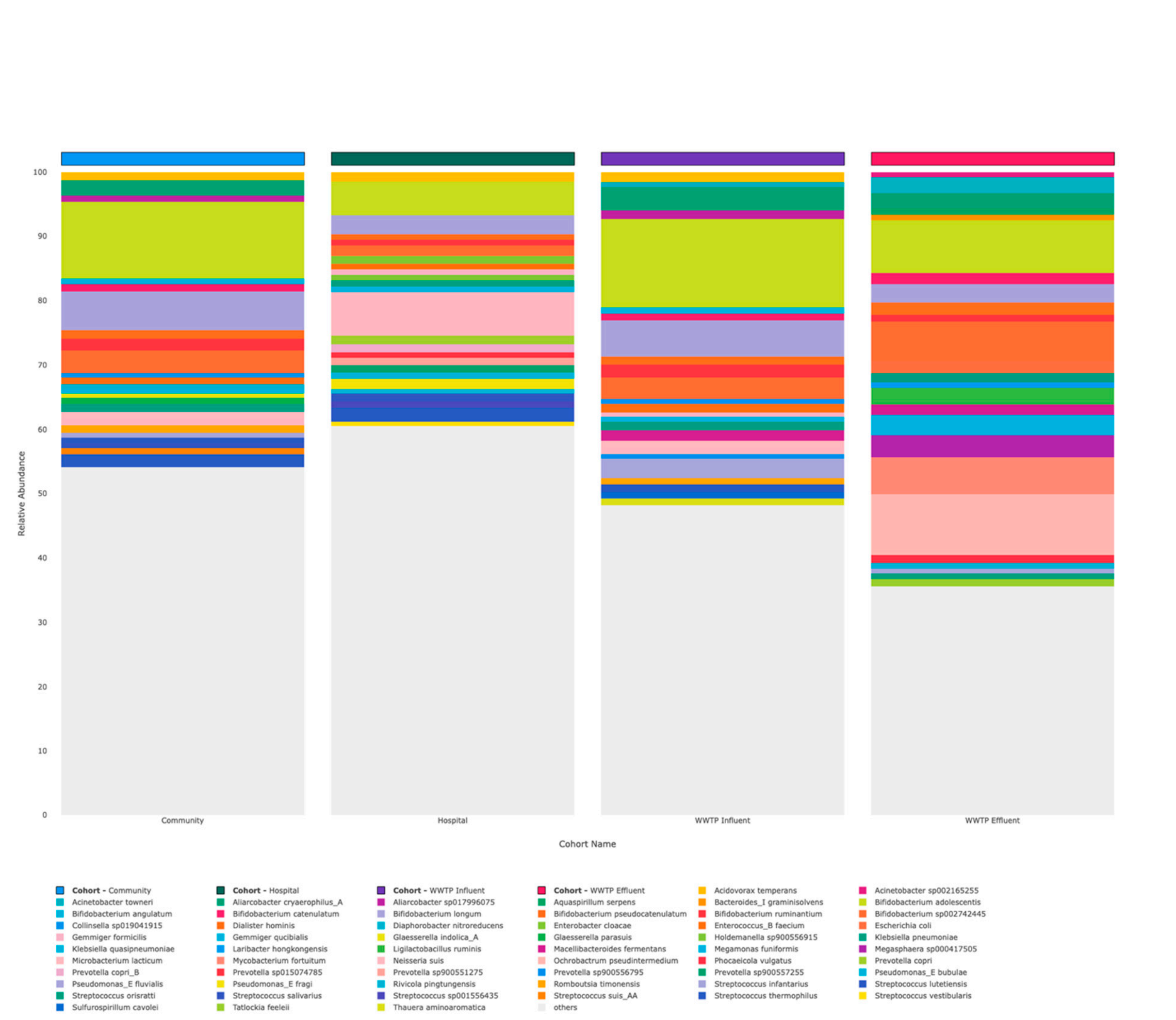

Figure S1. Relative abundance of the top 25 bacterial species across wastewater sources.

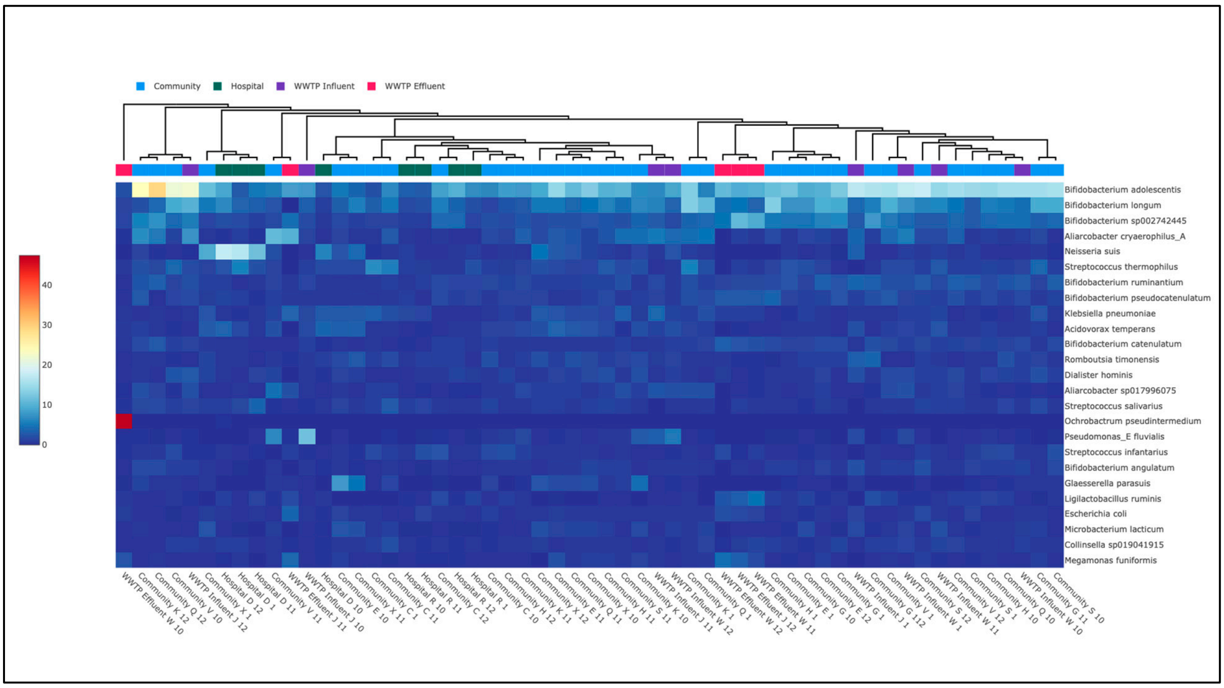

Figure S2. Heatmap showing the relative abundance of top bacterial species across wastewater samples, with hierarchical clustering based on ARG abundance profiles.

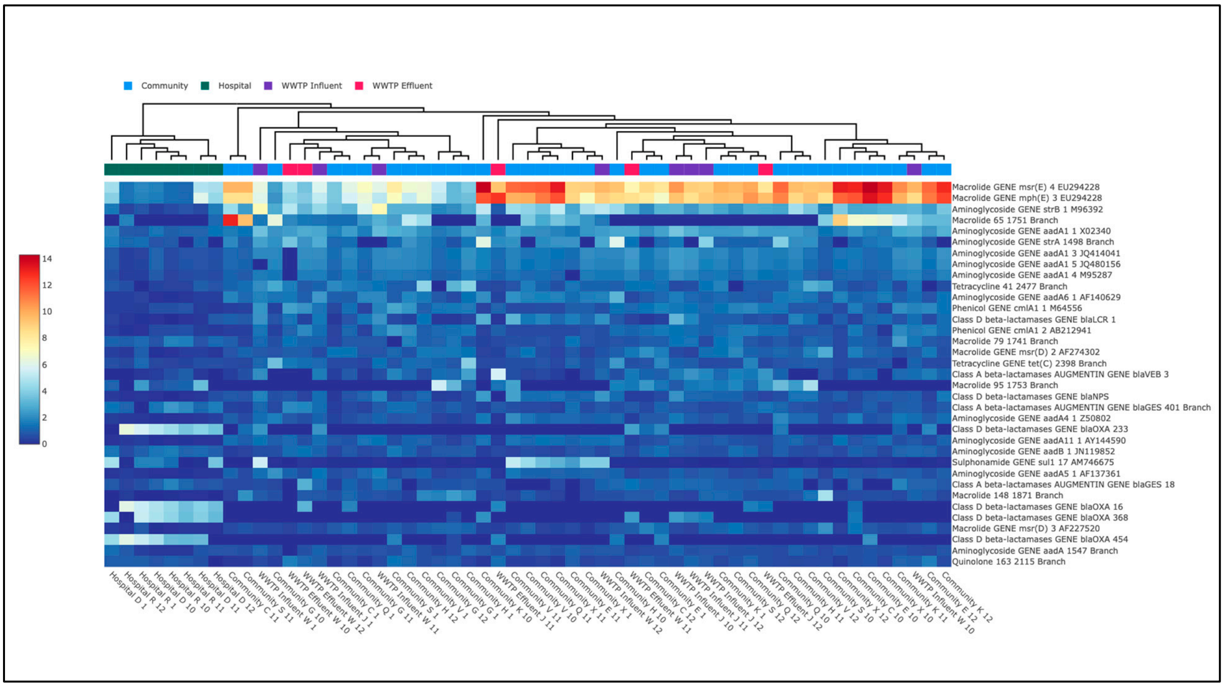

Figure S3. Heatmap showing the relative abundance of top ARGs across wastewater samples, with hierarchical clustering based on ARG abundance profiles.

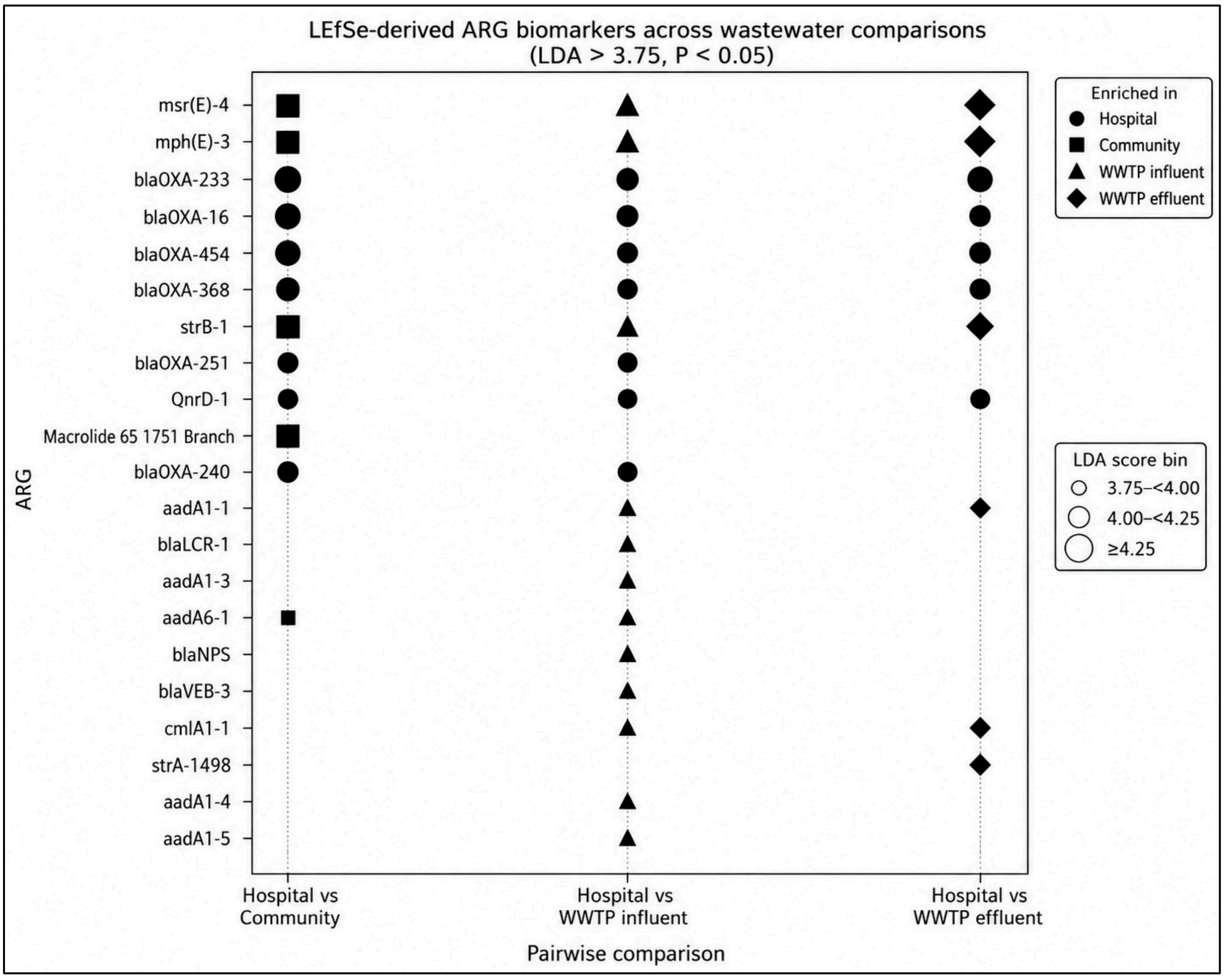

Figure S4. LEfSe-derived ARG across wastewater sources. Bubble plot showing antimicrobial resistance gene (ARG) identified by LEfSe analysis across pairwise wastewater source comparisons. Marker shape indicates the wastewater source in which each ARG was enriched, and marker size represents the LDA score bin. Only biomarkers meeting the significance threshold of LDA > 3.75 and $p < 0.05$ are shown.

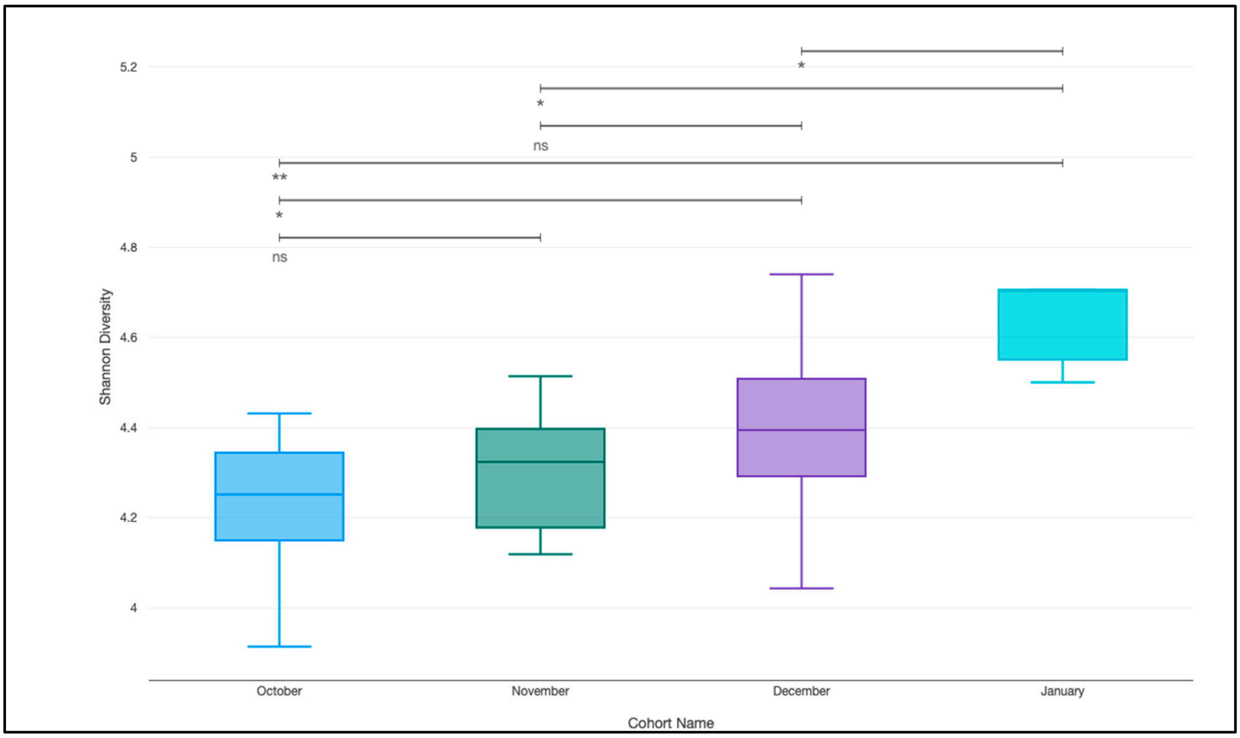

Figure S5. Temporal variation in ARG alpha diversity across sampling periods. Boxplots show Shannon diversity from October 2024 to January 2025, with points representing individual samples. Boxes indicate medians and interquartile ranges. Significance is denoted as *$p < 0.05$, **$p < 0.01$ and ***$p < 0.001$; ns, not significant.
